# Treatment of Gangrenous Wounds with Hot Oxygen

**Published:** 1915-05

**Authors:** 


**Treatment of Gangrenous Wounds With Hot Oxygen.** Vignat, Bull. et Mem. de la Soc. de Med. de Paris, Jan. 8, 1915. The oxygen, in a balloon, is delivered through a Gaiffe apparatus but the gasoline motor must not be used on account of danger of explosion. The rheostat tends to reach a white heat and must be handled carefully and there is also danger of setting fire to towels and dressings, and of applying too great heat to the parts. In certain cases, a caustic temperature is desired, in others a temperature of 55-60 C., producing hyperaemia, disinfecting and stimulating repair.

